# Perinatal Outcome of Vaginal Breech Delivery versus Caesarean Breech Delivery in a Tertiary Care Center

**DOI:** 10.31729/jnma.3697

**Published:** 2018-08-31

**Authors:** Rajendra Kumar Chaudary, Rajan Ghimire, Deepak Raj Kafle

**Affiliations:** 1 Department of Obstetrics and Gynecology, Pokhara Academy of Health Sciences, Kaski, Nepal; 2 Department of General Practice and Emergency Medicine, Tribhuvan University, Institute of Medicine, Kathmandu, Nepal; 3 Department of Obstetrics and Gynecology, Pokhara Academy of Health Sciences, Kaski, Nepal

**Keywords:** *breech presentation*, *cesarean breech delivery*, *vaginal delivery*

## Abstract

**Introduction:**

Breech delivery has always been matter of interest in obstetrics. Cesarean breech delivery has been preferred method of delivery. We aim to find out any differences in outcome between vaginal breech delivery and cesarean breech delivery in our setup.

**Methods:**

Data were collected from record book of Department of Gynaecology and obstetrics, Pokhara Academy of Health Sciences, Kaski, Nepal. Pregnant with breech presentation who had delivery in the centre from 2074 Baishak to 2074 chaitra were enrolled in the study. Data of 174 patients were analysed among which 74 underwent vaginal delivery for breech and 110 underwent cesarean breech delivery.

**Results:**

Only 1 (1.6%) of newborn delivered by vaginal route were admitted to NCU vs 17 (15.5% ) in cesarean group which was significant (odds ratio= 0.071, 95% C.I 0.009–0.574; p= 0.004). There was only one death of newborn which was delivered by vaginal route. Mean APGAR score at 1 and 5 minute in vaginal breech delivery was 6 and 7 and in cesarean breech delivery was 6 and 8.

**Conclusions:**

Though perinatal morbidity was more with cesarean breech delivery but further study with more sample size is needed before reaching conclusion.

## INTRODUCTION

Breech presentation is a longitudinal lie of the fetus with the caudal pole (buttock or lower extremity) occupying the lower part of the uterus and cephalic pole in the uterine fundus.^1^ There are three types of breech presentation. In the frank breech position (48 to 73%), both hips are flexed and both knees are extended. In the complete breech position (4.6 to 11.5%), both hips and both knees are flexed. In the incomplete breech position (12.4 to 40.5), one or both hips are not completely flexed.^2^ Prevalence of singleton breech deliveries in the hospital was 3.4%.^3^

There is increased risk to fetus born to breech because of cord compression between cervix and body during crowning; trauma to fetus from dystocia associated to after-coming shoulders, head and arms. So breech delivery is always been as a matter of study in obstetrics.

A study done in Lumbini medical college showed that out of 80 selected women with breech presentation, 42 of them had vaginal deliveries and 38 women had undergone caesarean section. The perinatal mortality was 4.8% and morbidity was 2% in vaginal breech deliveries. There was no significant difference of APGAR score in the two groups at any time. Similarly, there was no significant difference in perinatal morbidity and mortality in the two groups. Nulliparous women were more likely to deliver by cesarean section.^4^ Another study in Patan Academy of Health Sciences concluded that in well-selected cases, the neonatal outcome following assisted vaginal breech delivery and caesarean section may not be different.^5^

Adverse perinatal outcome in planned vaginal breech labor at term is associated with fetal growth restriction, oligohydramnios, previous cesarean delivery, gestational diabetes, nulliparity and epidural anesthesia.^6^ Women intending vaginal delivery had higher rates of neonatal morbidity (6.0% vs 2.1%), neonatal birth trauma (7.4% vs 0.9%), Apgar <4 at one minute (10.5% vs 1.1%), Apgar <7 at five minutes (4.3% vs 0.5%) and neonatal intensive care unit/special care nursery admissions (16.2% vs 6.6%) than those planning caesarean section.^7^ The term breech trial also concluded that perinatal mortality, neonatal mortality, or serious neonatal morbidity was significantly lower for the planned caesarean section group than for the planned vaginal birth group (17 of 1039 [16%] vs 52 of 1039 [50%]; relative risk 033 [95% CI 0-19-0-56]; p<0-0001). There were no differences between groups in terms of maternal mortality or serious maternal morbidity (41 of 1041 [3–9%] vs 33 of 1042 [3–2%]; 1–24 [0-79-1-95]; p = 0–35).^8^

As the neonatal outcome of planned cesarean breech delivery is good but in the situation like ours where pregnant lady arrives hospital in active stage of labor and vaginal trail is given. This study is aimed to find out the outcome of vaginal breech delivery in our setting.

## METHODS

It is a descriptive cross-sectional study conducted among the pregnant women with breech presentation at Department of Obstetrics and Gynecology, Pokhara Academy of Health Sciences, Kaski, Nepal. Secondary data were collected from the record of the department. Pregnant with breech presentation who had delivery in the center from 2074 Baishak to 2074 Chaitra were enrolled in the study. Sample size was calculated using standard formula.^9^ Data of 174 patients were analyzed among which 74 underwent vaginal delivery for breech and 110 underwent cesarean breech delivery. Data on age of patients, gravida, Parity, living child, abortion, previous neonatal death, period of gestation, mode of delivery (vaginal, cesarean delivery, instrumental) outcome (APGAR score at 1 min and 5 min, neonatal care unit admission, death, birth injuries) were collected. Collected data first entered into Microsoft excel first then analysed using SPSS 25.

Mean, frequencies and percentages were calculated for demographic data and categorical variables. Significance of categorical variable were analyzed using Chi square test. Multinomial logistic regression analysis was done to compare outcome among vaginal breech delivery and caesarean breech delivery.

## RESULTS

From 2074 Baishak to 2074 Chaitra there were 8687 deliveries out of which 184 (2.12%) were breech deliveries. Data of 184 patients were analyzed among which 74 underwent vaginal delivery for breech and 110 underwent cesarean breech delivery. Ten patients following vaginal breech delivery were excluded from studies because of missing data so 174 were included in the study. Frequencies and mean were used for categorical variables and chi-square test was used for significance test. Binary logistic regression was used for significance test of mode of delivery and its outcome. There was no significant difference between vaginal breech delivery group and cesarean breech delivery group in terms of age, parity and period of gestation.

In vaginal breech delivery group 77% were in age group 20–30 (mean = 24.2yrs; s.d = 4.22) and in cesarean delivery 67% belonged to 20–30 yrs (mean=24.4yrs; s.d=4.82). In both groups maximum was primipara (88% in Vaginal breech delivery group and 89% in cesarean delivery group) and term delivery (81% in vaginal group and 88% in cesarean group).

Mean APGAR score at 1 and 5 minute in vaginal breech delivery was 6 and 7 and in cesarean breech delivery was 6 and 8.

Binary logistic regression was used to compare outcome among different groups. Effect of age of mother, period of gestation, parity and mode of delivery were analyzed. The results were insignificant in case of age of mother, period of gestation and parity. However, in our study only 1.6% of newborn delivered by vaginal route were admitted to NCU vs 15.5% in cesarean group which was significant (odds ratio=0.071, 95% C.I 0.0090.574; P=0.004) (Table 2). There was only one death of newborn which was delivered by vaginal route but it was very small number so not included in analysis and need further study with large sample size. As there were no birth injuries in either of the group and none of the baby were delivered by instrumental method so both of these variables were not included during analysis.

**Table 1:** Frequency table of age group, parity and period of gestation in different mode of delivery.

Mode of Delivery									
			Vaginal delivery			Cesarean delivery			
	Count	Column %	Row %	Count %	Column %	Row N %	Chi square		
Age groups (years)	under 20	11	17%	34.4%	21	19%	65.6%	2.5951	0.2731
	20–30	49	77%	39.8%	74	67%	60.2%		
	30–40	4	6%	21.1%	15	14%	78.9%		
Category of parity	Primipara	57	89%	37.0%	97	88%	63.0%	0.0308	0.8605
	Multipara	7	11%	35.0%	13	12%	65.0%		
Period of gestation (weeks)	less than 37 week	12	19%	48.0%	13	12%	52.0%	1.5801	0.2087
	>37 weeks	52	81%	34.9%	97	88%	65.1%		

**Table 2:** Binary logistic regression analysis of comparison of outcome among vaginal breech and cesarean breech delivery

Outcome analysis										
		NICU		healthy		Chi-square	P	odds ratio		95% C.I
Age groups (years)	under 20	4	12.5%	28	87.5%					2.930
	20–30	12	9.8%	110	90.2%	0.193	0.908	1.059	0.383	
	30–40	2	10.5%	17	89.5%					
Category of parity	primipara	17	11.1%	136	88.9%	0.709	0.4	3.737	0.346	40.345
	multipara	1	5.0%	19	95.0%					
Period of gestation	less than 37 week	4	16.7%	20	83.3%	1.172	0.279	3.356	0.852	13.221
(weeks)	>37 weeks	14	9.4%	135	90.6%					
Mode of Delivery	vaginal delivery	1	1.6%	62	98.4%					
	cesarean delivery	17	15.5%	93	84.5%	8.263	.004	0.071	0.009	0.574

## DISCUSSION

With time and advent of safe surgical techniques more and more cesarean breech delivery are being performed worldwide. Cesarean breech delivery is considered safe mode of delivery for breech presentation.^7,8,10^ With more cesarean breech delivery being done, experience for vaginal breech delivery is decreasing so the risk for adverse outcome is increasing.

In the centers where vaginal breech delivery is performed more frequently outcome is still good in experienced and skilled hands.^11–14^ In our study there were 2.12% were breech deliveries which is similar to study conducted in Ethopia where prevalence was 3.4%.^3^ 15.5% newborn following cesarean breech delivery were admitted in neonatal care unit while 1.6% of newborn following vaginal breech delivery were admitted in NCU. As a subtotal population in NCU admission is less, superiority of vaginal delivery over cesarean delivery cannot made, further multicenter study with larger sample size is required.

## CONCLUSIONS

A total of 1.6% of newborn delivered by vaginal route were admitted to NCU vs 15.5% in cesarean group which was significant (odds ratio=0.071, 95% C.I 0.009-0.574; P=0.004). However, sample size was small so further study with larger sample size is needed.

## Conflict of Interest

**None.**
